# Understanding the Unfolded Protein Response in the Pathogenesis of Asthma

**DOI:** 10.3389/fimmu.2018.00175

**Published:** 2018-02-06

**Authors:** Prabuddha S. Pathinayake, Alan C.-Y. Hsu, David W. Waters, Philip M. Hansbro, Lisa G. Wood, Peter A. B. Wark

**Affiliations:** ^1^Priority Research Centre for Healthy Lungs, Hunter Medical Research Institute, University of Newcastle, Newcastle, NSW, Australia; ^2^Department of Respiratory and Sleep Medicine, John Hunter Hospital, Newcastle, NSW, Australia

**Keywords:** asthma, unfolded protein response, endoplasmic reticulum stress, innate immunity, inflammation

## Abstract

Asthma is a heterogeneous, chronic inflammatory disease of the airways. It is a complex disease with different clinical phenotypes and results in a substantial socioeconomic burden globally. Poor understanding of pathogenic mechanisms of the disease hinders the investigation into novel therapeutics. Emerging evidence of the unfolded protein response (UPR) in the endoplasmic reticulum (ER) has demonstrated previously unknown functions of this response in asthma development. A worsening of asthmatic condition can be brought on by stimuli such as oxidative stress, pathogenic infections, and allergen exposure. All of which can induce ER stress and activate UPR leading to activation of different inflammatory responses and dysregulate the innate immune functions in the airways. The UPR as a central regulator of asthma pathogenesis may explain several unknown mechanism of the disease onset, which leads us in new directions for future asthma treatments. In this review, we summarize and discuss the causes and impact of ER–UPR in driving the pathogenesis of asthma and highlight its importance in clinical implications.

## Introduction

Asthma is considered as one of the top five respiratory diseases in the world, and it affects 334 million people globally (1). The World Health Organization estimates that nearly 250,000 people die from asthma each year, worldwide (2). Asthma is a chronic inflammatory airways disease, in which clinical manifestations result from airway obstruction, airway hyperresponsiveness (AHR) and airway inflammation (3). It is associated with recurrent symptoms such as wheezing, dyspnea (shortness of breath), chest tightness, and cough. The asthmatic airway is characterized by an increased infiltration of eosinophils, neutrophils, macrophages, activated mast cells, and T helper cell type 2 (Th2) cells (4). The large airways manifest several structural changes that include a higher collagen deposition under the basal epithelium, increased airway smooth muscle, and an increased number of blood vessels (5). Asthma can be triggered by various stimulants such as indoor allergens (house dust mites), outdoor allergens (pollens and molds), tobacco smoke, chemical irritants, air pollution, virus infections, cold air, stressors causing emotions such as anger or fear, and physical exercise. Certain medications can also exacerbate asthma, such as aspirin and other non-steroid anti-inflammatory drugs, and beta blockers (6). Asthma is characterized with Th2 immune responses in a large proportion of patients. Th2 based cytokines, such as interleukin (IL)-4, IL-5, IL-9, and IL-13, are known to promote eosinophilic inflammation and immunoglobulin E (IgE) production by mast cells. IgE enhances the production of inflammatory mediators such as histamine, which triggers bronchospasm and mucus secretion from goblet cell, all of which are hallmark features of asthma (7, 8). The transcription factor GATA3 that promotes the expression of IL-4/-5/-13 from Th2 cells and facilitates the differentiation of naive T cells toward Th2 phenotype. GATA3^+^ T cells have been shown to be increased in the airways of stable asthmatic patients and were associated with increased levels of Th2 cytokines (9, 10). The alarmin IL-33 has also shown a role in promoting the differentiation of Th2 and type 2 innate lymphoid cells (11). In addition, recent studies have shown that Th17, Th9, and regulatory T cells (IL-10 and transforming growth factor-β (TGF-β) producing cells) also contribute to the pathogenesis of asthma (12–14).

The unfolded protein response (UPR) in the endoplasmic reticulum (ER) has been identified as a master regulator in several inflammatory diseases (15). It is an important adaptive response that provides protein translation and folding homeostasis, regulates immune responses to various exogenous stimuli such as allergens and pathogens, and serves as a decision point of flight-or-die response. The inflammation or cell death induced by UPR is a driving factor for neurodegenerative diseases (Alzheimer’s disease and Parkinson’s disease), metabolic diseases (type II diabetes), and inflammatory diseases (type I diabetes and inflammatory bowel disease), etc. (15). Manipulation of ER chaperon activity to regulate the protein folding and avoid protein aggregation has been used as a therapeutic approach for abovementioned diseases (15). While UPR has a decisive role in various molecular events, the role of UPR and how UPR dysregulation contributes to the pathogenesis of asthma remains unclear. The cells of the asthmatic airway, exposed to chronic inflammation with recurring cycles of damage and repair, are likely to be exposed to high levels of ER/UPR. Emerging evidence of *in vitro* and *in vivo* experiments further suggests a robust correlation of UPR in asthma development. Therefore, insights into the UPR in the regulation of asthma would be important to reveal unknown function in disease development. In this review, we discuss the roles of UPR with the aim to better understand the dynamics of UPR in asthma and potentially identify novel therapeutic approaches.

## ER Stress and Its Role in Immunity and Inflammation

### ER–UPR Signaling Pathways

The ER is mainly responsible for the biosynthesis, trafficking, and posttranslational modification of secreted and transmembrane proteins. The ER ensures release of properly folded proteins *via* secretory pathways, while improperly folded proteins degrade through ER-associated degradation (ERAD), or autophagy (16). ER homeostasis can be disturbed by physiological and pathological insults such as high protein demand, viral infections, environmental toxins, and inflammatory cytokines (17) resulting in a high proportion of misfolded or unfolded proteins in the ER. Accumulation of misfolded or unfolded proteins inside the ER leads to a series of adaptive mechanisms termed the UPR, which restores protein folding homeostasis (18). There are three transmembrane proteins that exist in the ER lumen that detect ER stress; inositol-requiring protein 1α (IRE1α), protein kinase RNA-like endoplasmic reticulum kinase (PERK), and activating transcription factor 6α (ATF6α). These transmembrane proteins act as sensors of ER stress and are held in check by physical interaction with the chaperone protein immunoglobulin binding protein (BiP). Once ER stress is induced, the UPR is triggered to restore protein folding homeostasis by controlling protein translation, increasing folding capacity, or activating ERAD. If ER stress cannot be resolved, then apoptosis is triggered to remove stressed cells and avoid harmful effects of misfolded proteins (19).

**Figure 1:**
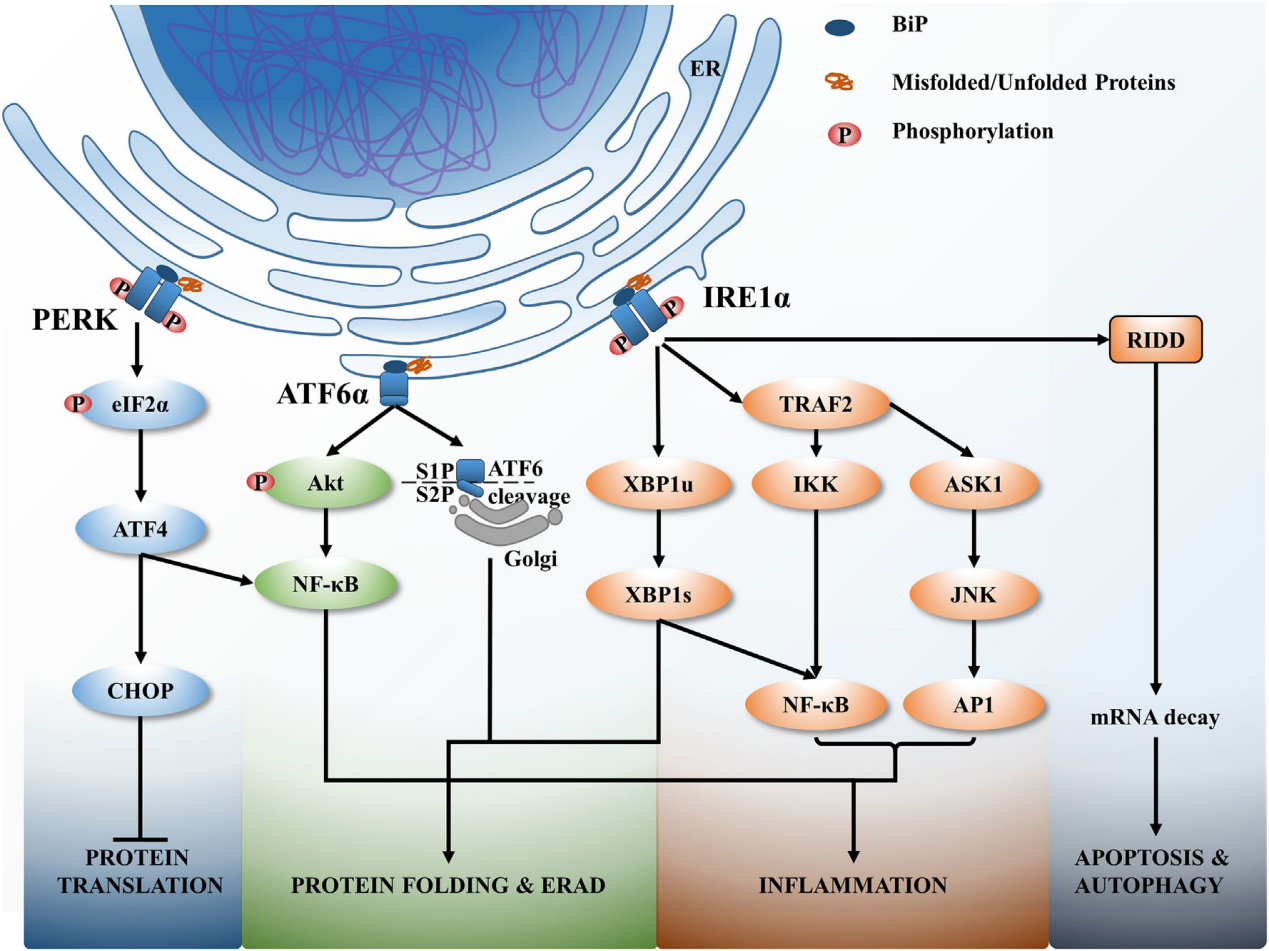
The unfolded protein response (UPR) signaling pathways. Upon endoplasmic reticulum (ER) stress, ER resident chaperon BiP dissociates from ER transmembrane sensors and binds with misfolded or unfolded proteins that accumulate within the ER. BiP dissociation activates UPR to correct the protein folding or if unresolved leads to cell death. The activation of three main signaling branches of the UPR; protein kinase RNA-like endoplasmic reticulum kinase (PERK), activating transcription factor 6α (ATF6α), and inositol-requiring protein 1α (IRE1α) lead to attenuate protein translation, increase the misfolded protein degradation (ER-associated degradation), increase protein folding *via* activating numerous protein chaperons, induce various inflammatory responses and if unresolved cell apoptosis and autophagy.

Activating transcription factor 6 is a leucine zipper protein and a type II ER transmembrane protein. When misfolded proteins accumulate inside the ER, BiP (also known as GRP78 and HSPA5) dissociates from ATF6 allowing it to interact with misfolded proteins (20). Consequently, ATF6 translocates into the Golgi and is processed by proteases S1P and S2P (Figure 1). As a result, the N-terminal cytosolic domain of ATF6 translocates to the nucleus and induces transcription of a number of UPR genes, including acute phase response (APR) associated genes (21).

Inositol-requiring protein 1 is a type 1 ER transmembrane protein that is activated by the dissociation of BiP from its luminal domain. Accumulated misfolded proteins associate with IRE1α resulting in oligomerization and autophosphorylation of the kinase domain and activation of RNase domain of IRE1α (Figure 1). This induces splicing of X-box binding protein 1 (XBP1) mRNA to express transcriptional factors and upregulates UPR genes related to protein folding, protein secretion, and ERAD (22). Chronic persistence of ER stress activates IRE1 to interact with tumor necrosis factor associated factor 2 (TRAF2) leading to the activation of downstream apoptosis signal regulating kinase 1 (ASK1)–JNK signaling pathway to trigger apoptosis (23).

Protein kinase RNA-like endoplasmic reticulum kinase is the third arm of the UPR and is a type I ER transmembrane protein activated by recognizing misfolded proteins inside the ER, followed by oligomerization and autophosphorylation. This activation triggers phosphorylation of eukaryotic initiation factor 2α (eIF2α) that attenuates protein translation to control the protein load of the ER (Figure 1). In addition, eIF2α activates the translation of eIF2α-activating transcription factor 4 (ATF4) that in turn activates transcription of several UPR related genes, including ERAD, that then leads to autophagy, apoptosis, and redox homeostasis (24).

Recognition of ER stress by the aforementioned ER transmembrane proteins within the ER lumen has been described (19). Originally, it was believed that dissociation of BiP from ER transmembrane sensors during ER stress initiates the UPR (25). However, recent studies indicate that BiP is not the only chaperone initiating the UPR for all ER transmembrane sensors (26). This model of UPR activation showed that the unfolded proteins directly bind to either IRE1-α or PERK. This interaction of unfolded proteins with these luminal domains induces conformational changes and oligomerization to also initiate the UPR. It is likely that direct binding of unfolded or misfolded proteins to IRE1/PERK determined by the nature of the protein as IRE1 is intolerance for acidic residues and favorable for basic and hydrophobic residues (27). The third model of UPR activation proposes that both BiP dissociation and direct binding of peptides cause the UPR activation (28), which could be a more reliable and efficient way of handling unfolded/misfolded proteins as it can detect wide range of unfolded proteins *via* BiP or direct binding.

### UPR and Immunity

The UPR is involved in regulating several innate and adaptive immune pathways. Classically, the UPR was considered an adaptive response triggered by ER stress to restore the protein folding inside the ER. However, recent findings have revealed a plethora of signaling pathways associated with UPR, which have been implicated in various developmental disorders, and immune responses.

Although in most of cells UPR signaling cascades activate in conditions of ER stress, in some cells the UPR is activated as a normal cellular process regardless of ER stress (29). For instance, the IRE1–XBP-1 signaling pathway has been shown to play an essential role in the differentiation of B cells to plasma cells. Knockdown of XBP-1 in B cells drastically minimizes immunoglobulin production. The plasma cell population in lymph nodes, spleen, bone marrow, or the lamina propria of the gut were all reduced in the absence of XBP-1, suggesting XBP-1 is an essential molecular switch for plasma cell generation (30). Further, the absence of XBP1 has been shown to enhance the expression of IRE1, which triggers regulated IRE1-dependent decay (RIDD). This activation cleaves the mRNA of secretory μ chains in plasma cells and consequently reduces IgM levels (31). In addition, in both conventional and plasmacytoid dendritic cells, XBP-1 play a central role in cell development and survival (32). Lymphoid chimeras lacking XBP-1 demonstrated decreased numbers of both conventional and plasmacytoid DCs with reduced survival both at baseline and in response to TLR signaling. By contrast, overexpression of XBP-1 in hematopoietic progenitors rescued and enhanced DC development (32). IRE1–XBP-1 axis has also been shown to be critical in the biology of CD8α^+^ conventional DCs (cDCs). A deficiency of XBP-1 affects antigen presentation, phenotypes and ER homeostasis of CD8α^+^ cells but curiously not in the closely related CD11b^+^ cDCs, highlighting the importance of XBP-1 in a subset of specific DCs (33). Brunsing et al. demonstrated that IRE arm is activated in the CD8^+^ cytotoxic T-cell population; however, it was not detected in mature CD4^+^ T-cell populations, suggesting that XBP1 splicing is important during early stages of both B and T cell development (34). In addition, XBP-1 splicing is upregulated in antigen-specific CD8^+^ T cells during viral and bacterial infection. XBP-1 splicing enhances the level of killer cell lectin-like receptor G1 in CD8^+^ T cells during viral infection and contributes to the differentiation of end-stage effector CD8^+^ T cells (35). Therefore, UPR activation is a requirement of several components of immune system and dysregulation of UPR could results various immunological disorders.

### ER Stress Regulates Inflammation

The ER stress has been shown to be an important regulator for numerous chronic diseases including: inflammatory bowel diseases, atherosclerosis, type II diabetes, cancer, liver diseases such as non-alcoholic fatty liver disease, alcoholic liver disease, hepatitis C virus (HCV)/HBV infection, and neurodegenerative diseases such as Alzheimer’s disease, Parkinson’s disease, and amyotrophic lateral sclerosis (36–39). ER stress regulates inflammation by activating numerous inflammatory signaling pathways, and increased inflammation can reciprocally induce the ER stress (40). These inflammatory signaling pathways converge at NF-κB, the master transcriptional factor of pro-inflammatory cytokines. In response to ER stress, IRE1α interacts with TRAF2 and recruits IκB kinase, which leads to phosphorylation and degradation of IκB and the subsequent nuclear translocation of NF-κB. The IRE1α–TRAF2 complex can also recruit ASK 1, and subsequent activation of JNK and AP1 also interacts with nuclear NF-κB and induce the expression of pro-inflammatory cytokines (41). In addition, the spliced XBP1 in IRE1α arm can directly induces the transcription activation of pro-inflammatory genes such as IL-6 and TNF (42).

The PERK–eIF2α branch of UPR activates NF-κB *via* an alternative mechanism. Activation of the PERK–eIF2α affects protein translation, resulting in downstream effects of the NF-κB to IκB ratio, favoring NF-κB-dependent transcription (43). In addition, the ATF4, downstream of PERK activation, directly acts as a transcription factor for IL-6 (44). The ATF6α branch of the UPR also activates NF-κB through an alternative mechanism, *via* phosphorylation of the serine/threonine kinase Akt (Akt) (PI3K pathway) and degrading NF-κB inhibitor, IκB (Figure 1) (45, 46). In addition, ATF6α can trigger an APR, which amplifies the pro-inflammatory response upon infection (40).

Activation of NLRP3 inflammasome by the UPR has been reported (47–49). Chronic irremediable ER stress has been shown to induce thioredoxin-interacting protein *via* IRE1α to activate NLRP3 inflammasome and promote apoptosis (49). Moreover, Bronner et al. demonstrated that ER stress modulates the inflammasome by initiating mitochondrial damage *via* IRE1α pathway. IRE1α induces the release of mitochondrial danger-associated molecular patterns by NLRP3–caspase2–bid signaling, which activates inflammasome (50). By contrast, Menu et al. showed that ER stress induced NLRP3 inflammasome is independent of UPR (47). Absence of UPR effectors IRE1α, PERK, ATF6, or CHOP did not affect the activation of NLRP3 by ER stress. However, it shared the same requirement for reactive oxygen species (ROS) production and potassium efflux to activate the NLRP3 inflammasome. Therefore, they suggest ER stress activates NLRP3 inflammasome *via* mitochondria without the involvement of the classical UPR signaling cascade (47). These variable observations could be due to different severity and longevity of ER stress exposure in different cell types used in different experiments. Therefore, further investigations would be important to understand how different conditions of UPR regulate inflammasome.

The link between ER–UPR and the induction of type I and III IFNs is not as clear. UPR-inducing agents tunicamycin and thapsigargin treatment do not induce the expression of these IFNs. However, when tunicamycin or thapsigargin treated cells were then treated with lipopolysaccharide (LPS) or a synthetic RNA poly I:C, IFN-β production was substantially increased. This increase was also accompanied with inductions of pro-inflammatory cytokines including IL-6 and TNF-α (51, 52).

This was in contrast with another study that showed that ER stress was sufficient to activate IRF3 phosphorylation. ER stress/UPR-mediated phosphorylation of IRF3 appears to be dependent on the type of the ER stress (53, 54). Dysregulated Ca^2+^ signaling (by thapsigargin, an SERCA pump inhibitor) appears to activate intracellular DNA sensing STING–TBK1–IRF3 pathway, whereas tunicamycin (N-linked glycosylation inhibitor) activates IRF3 in a STING-independent, but ATF6-dependent pathway (54). The exact molecular signaling pathways that connects the UPR to STING is currently unclear, but may involve cGAS–cGAMP–STING pathway if ER stress/UPR is induced by DNA viruses. Therefore, this emphasizes the dynamics of UPR in response to various stimuli, and may potentially induce different arms of UPR in response to different stimuli.

## ER Stress in Asthma

### Chemical Chaperones Inhibit ER Stress and Control Asthma Pathogenesis

Ova-induced mouse models of allergic asthma, have implicated ER stress playing a role along with heightened NF-κB-mediated inflammation in the airways (55, 56). Mice sensitized with ovalbumin (OVA) and LPS, followed by another challenge with OVA (OVA_LPS_–OVA mice), showed enhanced ER stress/UPR markers; BiP, CHOP, ATF6α, XBP1 and p-eIF2α in lung tissue, increased infiltration of inflammatory cells, and enhanced inflammatory cytokines in BAL fluid. Interestingly, 4-phenylbutyric acid (4-PBA) treatment, a chemical chaperone that inhibits ER stress/UPR, reduces NF-κB activity, inflammatory cytokines production (IFN-γ, IL-4, IL-5, IL-13, TNF-α, IL-1β, and IL-17), inflammatory cell infiltration in the lung, TLR4 expression, and bronchial hyperresponsiveness in OVA_LPS_–OVA mice. Induction of ER stress by tunicamycin exposure enhances asthma like responses in OVA_LPS_–OVA mice, suggesting that accumulation of misfolded/unfolded proteins in ER could be aggravating the asthmatic condition. In addition, upregulated UPR in human asthmatic patient samples, specifically in BALF and PBMC, was also identified (55). These results suggest that ER stress may play an important role in asthma pathogenesis, at least in part by regulating NF-κB activity. Concluding the overall results with neutrophilic asthma mice models, authors suggest that pharmacological interventions such as 4-PBA may prove to be a better strategy to control neutrophilic-dominant severe asthma (55).

A UPR was also seen to be significantly increased in the lungs of OVA sensitized and challenged mice (OVA–OVA mice) (56). This UPR was characterized by the initial increase in ER resident chaperones, such as GRP78, GRP94, and subsequent expression of the maladaptive UPR marker CHOP. Interestingly, the asthma like phenotype characterized by histological/biochemical markers and AHR become more prominent under maladaptive UPR conditions, indicating the presence of chronic ER stress in asthma. Pretreatment (preventive regimen) in mice with chemical chaperones [glycerol, trehalose, and trimethylamine-*N*-oxide (TMAO)] that increase the protein folding capacity of the ER reduced the maladaptive UPR, resulting in less severe AHR, inflammation, mucus metaplasia, and collagen deposition. Moreover, the therapeutic application of chaperones (4-PBA and TMAO) with ongoing allergen challenge, effectively controlled the maladaptive UPR response and abovementioned asthmatic features. This highlights the potential of targeting ER/URP as therapeutic intervention for asthma (56).

### Upregulated ORMDL3 Activity Induces the ER Stress in Asthma

**Figure 2:**
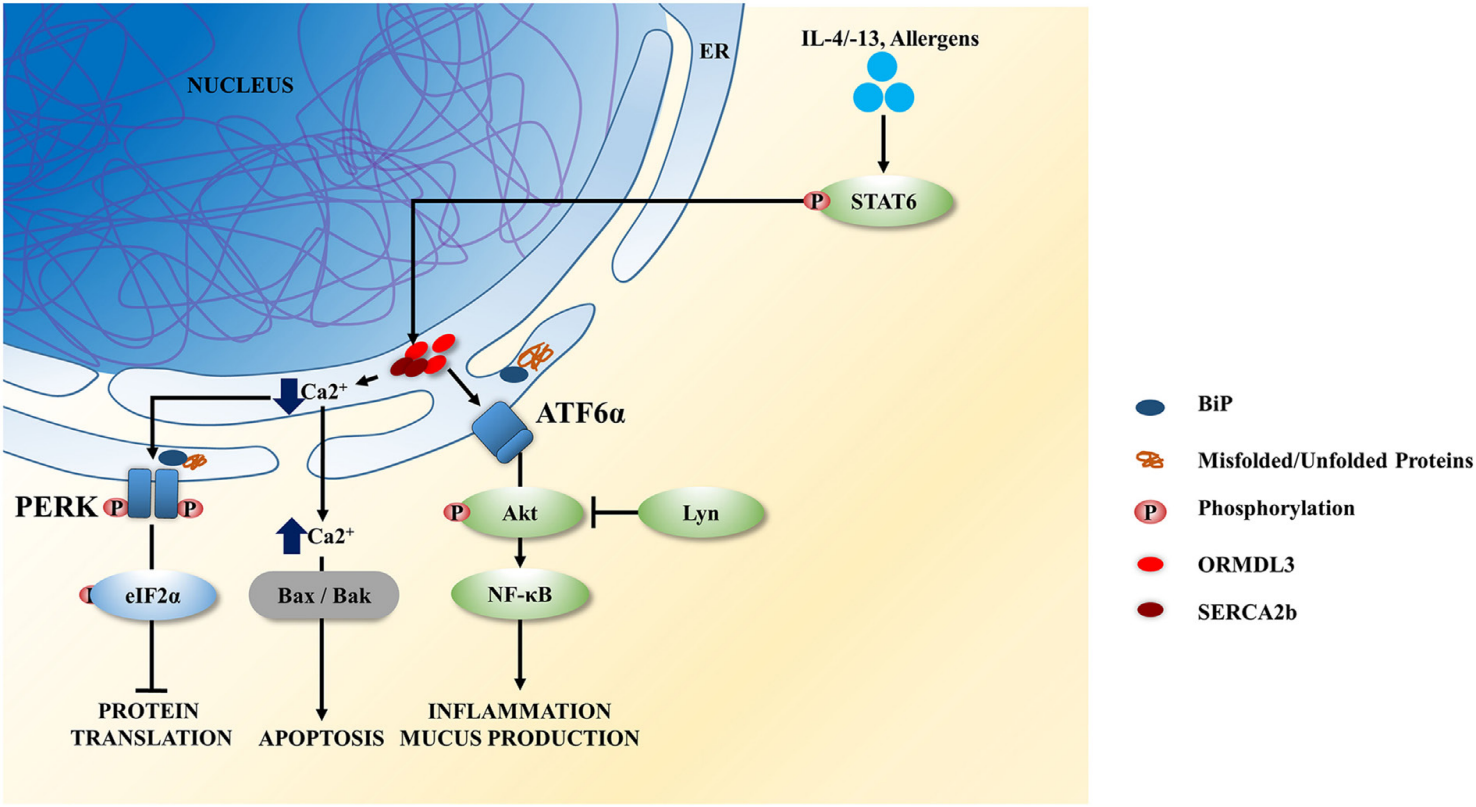
ORMDL3 induced unfolded protein response (UPR) signaling in airway epithelium. ORMDL3 is highly induced by allergens and interleukin (IL)-4/13 *via* STAT6 activation. Upregulated ORMDL3 negatively regulates the Ca^2+^ influx in endoplasmic reticulum (ER) by inhibiting the sarco-endoplasmic reticulum Ca^2+^ ATPase 2b (SERCA2b) activity and reducing the uptake of cytoplasmic Ca^2+^ into the ER. This results in activation of the protein kinase RNA-like endoplasmic reticulum kinase (PERK) signaling branch of the UPR leading to the attenuation of protein translation. SERCA dysfunction increases the cytoplasmic Ca^2+^ level and thereby and thereby initiate cellular apoptosis *via* Bax/Bak signaling. Increased ORMDL3 and Lyn kinase activity have also been show to activate the ATF6 branch of UPR resulting in increases of inflammatory cytokines secreted and mucus hypersecretion.

ORMDL3 is an ER resident transmembrane protein that can be induced by allergens, IL-4/-13 *via* STAT6 activation (57) and has been associated with asthma susceptibility (58–60). ORMDL3 negatively regulates calcium (Ca^2+^) influx in the ER and promotes UPR activation. ORMDL3 directly interacts and inhibits the activity of sarco-endoplasmic reticulum Ca^2+^ ATPase 2b, impeding the uptake of cytoplasmic Ca^2+^ into ER, therefore leading to increased PERK–eIF2a and UPR activity (Figure 2) (61). The reduced activity of SERCA by ORMDL3 may contribute to airway remodeling in asthma as Ca^2+^ homeostasis in airway smooth muscles is important for airway remodeling, regulating cell proliferation, cell spreading, and pro-inflammatory cytokine release (62). Overexpression of *ORMDL3* results in a decrease in Ca^2+^ levels in the ER, leading to increased cytosolic Ca^2+^, which in turn activates apoptosis, *via* Bax and Bak (Figure 2) (63). Cells deficient in both Bax and Bak are resistant to ER stress/UPR-mediated apoptosis. The asthmatic bronchial epithelium has been shown to have increased expression of ORMLD3, and this is associated with dysregulated Ca^2+^ homeostasis and increased ER stress/UPR (64).

### ER Stress and Mucus Hypersecretion in Asthma

Asthma is characterized by mucus hypersecretion. The mucin gene, *MUC5AC*, a low-charge glycoform of *MUC5B*, and *MUC2* are highly expressed in the asthmatic epithelium (65). Martino et al. demonstrated that the ER stress transducer IRE1-β is crucial for mucin production in the human primary bronchial epithelial cells. This IRE1-β-dependent mucin production relies on XBP-1 splicing and transcriptional activation of anterior gradient homolog 2, which is a key initiative factor in airway and intestinal mucin production. Intriguingly, the expression of IRE1-β in human asthmatic epithelium is significantly higher than the non-asthmatic epithelium suggesting that the increased IRE1-β expression in asthma is associated with increased mucus production and goblet cell metaplasia (66). Knockdown of *IRE1-*β considerably reduced the number of goblet cells, *MUC5B/MUC5AC* expression, and IL-13 expression in mouse OVA model. IRE1-β does not regulate BiP, ATF4, or CHOP activity, but it specifically enhances the XBP-1 splicing and this regulates the transcriptional activity of mucin genes. Drugs specifically targeting IRE1-β activity may be potential anti-mucus therapeutic agents, to reduce mucus overproduction in asthma and other inflammatory airway diseases (66).

Kaempferol is a natural plant-derived flavonol, which is an antioxidant and a possible cancer treatment. Kaempferol has been shown to act as an inhibitor of ER stress induced mucus production in the airway epithelium (67). An epithelial cell line (BEAS-2B cells) when treated with tunicamycin or TGF-β showed increased UPR and *MUC5AC* induction. Similarly, OVA sensitized BALB/c mice showed goblet cell hyperplasia, mucus hypersecretion, enhanced XBP1, and IRE1-α activity suggesting the direct link between UPR and mucin upregulation. Interestingly, treatment with kaempferol significantly reduced the ATF6 and IRE1-α branches of the UPR and blocked IRE1α–TRAF2–JNK activation. As a result, this impaired the XBP1 splicing and transcriptional activation of *MUC5AC* (67).

Upregulation of Lyn kinase activity has been shown to suppress mucus hypersecretion by downregulating the IL-13 induced ER stress in a mouse OVA model. Lyn overexpressed transgenic mice sensitized and challenged with OVA displayed reduced airway inflammation mucus production, goblet cell population and UPR (68). Similarly, Lyn kinase overexpression in IL-13 treated bronchial epithelial cells also showed reduced UPR, inflammation, and mucin markers. These effects are comparable to the effect of 4-PBA, which is a well-known ER stress inhibitor suggesting that Lyn kinase worked similarly to 4-PBA to alleviate ER stress. Lyn overexpression or 4-PBA treatment reduces the activation of PI3K p85α and Akt and consequently limits the UPR. Reduced UPR downregulates NF-κB p65 expression and its transcriptional activation of *MUC5AC* and subsequent mucus production (68). Thus, increased UPR in asthma models, particularly *via* the IRE1 pathway, may contribute to the heightened inflammatory response and mucus hypersecretion. Inhibition of IRE1 therefore poses as a potential therapeutic option to reduce these two important hallmark features of asthma.

### ER Stress Is Evident in Allergic Lung Inflammation in Fungal Asthma

Airborne fungal species such as *Alternaria, Aspergillus, Cladosporium*, and *Penicillium* may trigger worsened asthma. It is reported that globally more than 6.5 million people have severe asthma with fungal sensitizations, up to 50% of adult asthmatics attending secondary care have fungal sensitization, and 4.8 million adults have allergic bronchopulmonary aspergillosis (69–71). A mouse model was used to demonstrate that *Aspergillus fumigatus* (AF) could induce eosinophilic steroid refractory asthma associated with increased ER stress (72). AF exposed mice and murine primary tracheal epithelial cells demonstrated an increased expression of UPR markers; GRP78, CHOP, p-IRE1-α, p-eIF2α, XBP1, ATF4, and enhanced PI3K-δ activity. Interestingly, inhibition of PI3K-δ reduced the AF induced UPR and inflammation. Treatment with 4-PBA significantly reduced the AF induced lung inflammation. These results suggest that AF exposure in lungs induces allergic inflammation by activating PI3K-δ pathway and UPR. Similar to what was seen with OVA and LPS, enhanced UPR upregulates the nuclear translocation of NF-κB ultimately increasing the inflammatory response (72). *Alternaria*-induced allergic airway disease has been associated with ORMDL3 activity. Upon *Alternaria* exposure, ORMDL3 activates the UPR *via* the ATF6 pathway by increasing the transcription of ER-associated protein degradation pathway specific genes (Edem-1). This in turn upregulates the expression of the inflammatory cytokine IL-6. *ORMDL3* deficiency protected mice from developing *Alternaria-*induced AHR, the cellular stress, while overexpression of *ORMDL3* enhanced allergic symptoms (73).

### Allergens Induce ER Stress in the Airway Epithelium

House dust mite (HDM) is a frequent and important, trigger of allergic asthma and exposure results in activation of innate immune responses, the production of cytokines that can regulate subsequent activation of T cells, mucus metaplasia, inflammation, AHR, and fibrosis (74, 75). HDM exposure to primary nasal epithelial cells, BECs or mice challenged with HDM elicited a robust ER stress/UPR (76, 77) *via* increasing the phosphorylation of IRE1 (p-IRE), and expression of ER chaperone BiP, GRP94, and ERp57. HDM also activates the ATF6α pathway, its downstream transcriptional effector CHOP, caspase-3 activity and consequently leads to apoptosis. Knockdown of ATF6α or ERp57 significantly reduced HDM-induced apoptosis by decreasing CHOP expression, caspase-3 activity, and partially reduced airway inflammation and AHR. However, GRP78/GRP98 pathways of UPR were not affected by ATF6α/ERp57 knockdown. Similarly, human asthmatic airway epithelial cells were shown to have increased activity of ERp57, which was further elevated following HDM exposure. In murine model of HDM sensitized allergic asthma, elevated ERp57 in the epithelial cells was associated with heightened airway inflammation, AHR, and fibrosis (by increased collagen depositions) (76).

Chemical chaperones have been tested to control the UPR and reduce HDM-induced asthmatic features. Tauroursodeoxycholic acid (TUDCA) is an inhibitor of ER stress/UPR and is clinically used in the treatment of cholelithiasis and cholestatic liver diseases (78). Siddesha et al. demonstrated that TUDCA administration to HDM challenged mice markedly attenuates the HDM-induced airway inflammation, mucus metaplasia, ER stress markers, AHR, and airway fibrotic remodeling (79). While the molecular mechanisms of TUDCA-mediated reduction of ER–URP in asthma is unclear, a study showed that TUDCA inhibited H_2_O_2_-induced PERK and IRE1 signaling, which led to reduced ROS production and apoptosis in a mouse models of ischemia (80).

### Cigarette Smoke Induces ER Stress in Airway Epithelium

Exposure to cigarette smoking is a significant factor in the development of poor asthma control and acute exacerbations (81). Cigarette smoke results in oxidative damage, inducing the release of pro-inflammatory cytokines and epithelial permeability, all of which contributes to the exacerbations and worsening of airway inflammation in asthma.

Cigarette smoke has been shown to activate the PERK–eIF2α pathway, CHOP induction, and upregulation of BiP (82, 83). In addition, cigarette smoke-induced phosphorylation of ERK1/2 and activation of NF-κB that leads to the induction of several inflammatory signaling pathways (84). It is currently unclear if cigarette smoke causes heightened UPR and contributes to the pathogenesis of asthma, further investigations are needed to delineate the roles of cigarette smoke-induced UPR in asthma pathogenesis.

### Virus-Induced UPR in Asthma

Viral infections are known to induce ER stress/UPR. Viruses hijack the host cellular machineries to produce viral proteins; this places a heavy demand on the protein folding mechanisms in ER, resulting in high ER stress and UPR (85). Hassan et al. showed that influenza A virus (IAV) infection in human tracheobronchial epithelial cells induces ER stress and the IRE1α arm of UPR (86). It increased the expression of CHOP and XBP1 splicing but did not affect PERK or ATF6α signaling, therefore leading to the activation of inflammation and apoptosis, but not protein translation inhibition and ERAD. Treatment with TUDCA or IRE1α inhibitor significantly reduced viral protein synthesis and viral replication (86). This indicates that enhanced ER stress is favorable for IAV infection.

Activation of PERK and consequent UPR by vesicular stomatitis virus and HCV has been shown to result in degradation of interferon-alpha/beta receptor alpha chain (IFNAR1) in a ligand-independent manner. Infection activated PERK and increased ligand and Jak-independent phosphorylation of IFNAR1, resulting in IFNAR1 ubiquitination and degradation. This led to inhibition of IFN signaling and increased viral replication (87).

As PERK activation results in the arrest of protein translation, viruses such as IAVs have evolved to inhibit PERK activation to promote viral protein production. IAV produces a non-structural 1 protein that not only inhibits type I and III IFN production (88), but has also been shown to activate p58IPK, an inhibitor of pIF2α phosphorylation and activation (89–91). NS1–p58IPK interaction therefore suppresses PERK-mediated inhibition on protein translation, and it is likely that the asthmatic bronchial epithelial cells are more vulnerable to the immunomodulatory effect of NS1. Picornaviruses, such as rhinovirus, however, have been suggested to benefit from autophagy because they require double-membraned vesicles such as autophagosomes as sites of RNA replication. Activation of IRE1α and autophagy by these viruses such as rhinoviruses (RVs) is therefore likely to promote their infection and replication (92) in asthmatic bronchial epithelium.

Individuals with asthma are highly vulnerable to the effects of viral infections such as RV and IAV, and virus-induced exacerbations causes exaggerated airway inflammation, mucus hypersection and AHR. Despite heightened inflammatory response, asthmatic patients have been shown to have impaired antiviral response, particularly the production of type I and III IFNs, and apoptosis (93, 94). The molecular mechanisms underpinning these abnormal immune responses in asthma are currently unclear, and UPR could be at the core of this imbalanced immune responses. In asthmatic bronchial epithelium, increased UPR may result in increased ATF6 and IRE1-mediated NF-κB activation and inflammation, while activated PERK leads to IFNAR1 ubiquitination and degradation, thereby reducing antiviral responses. This could prime the epithelial microenvironment to be more susceptible to viral infections.

Indeed, allergens such as HDM and cigarette smoke extracts have all been shown to activate the UPR in the airways. As asthmatic bronchial epithelial cells have increased expression/activity of ATF6α and XBP1, it is possible that asthmatic bronchial epithelium is primed to respond more vigorously in terms of inflammation. ORMDL3 expression has also been found to be increased in asthmatic bronchial epithelial cells, contributing to the enhanced ATF6α-driven inflammatory responses while increasing protein folding capabilities to RV infection (58–60). PERK-activation-mediated IFNAR1 degradation could contribute to impaired antiviral immunity in asthma during viral infection (87, 95). Nevertheless, molecular mechanisms of the impaired antiviral immunity in asthma remain to be determined.

## ER Stress/UPR Regulates Oxidative Stress and Mitochondrial Function in Asthma

Oxidative stress is heightened in the asthmatic airways (96–99). Endogenous and exogenous ROS such as superoxide anion, hydroxyl radical, hypohalite radical, and hydrogen peroxide, and reactive nitrogen species (RNS), such as nitric oxide, peroxynitrite, and nitrite, play a major role in the airway inflammation (100). Activated airway inflammatory cells and resident airway cells can equally contribute the pool of ROS production (101, 102).

The ER provides a unique oxidizing environment for protein folding. During disulfide bond formation, ROS are produced inside the ER, and it has been suggested that oxidation of cysteine residues during disulfide bond formation may significantly contribute to oxidative stress (103). Accumulation of unfolded (due to high demand) or misfolded (due to oxidation) proteins inside the ER lumen can produce large amount of H_2_O_2_ and can lead to depletion of glutathione (GSH) that is essential for redox homeostasis (104). In addition, the mitochondria produce more ROS by taking up Ca^2+^ released from the ER under stress. Excessive Ca^2+^ results in the release of cytochrome *C* from the mitochondria that then inhibits complex III of electron transport chain (ETC), leading to increased production of ROS. Moreover, Ca^2+^ stimulates the Krebs cycle dehydrogenases and enhances the production of ROS. It activates nitric oxide synthases, which disrupt the ETC and produce ROS (105, 106). Activation of CHOP as a proapoptotic regulator of the UPR also increases ROS by upregulating Ero1α, an oxidoreductase that relays disulfide bonds to protein disulfide isomerase (107). Activation of the PERK arm of the UPR subsequently activates ATF4 and NRF2, both transcription factors that trans-activate antioxidative stress response genes, including SODs, hemeoxygenase-1, glutathione transferase, and uncoupling mitochondrial protein 2 (108). Treatment with antioxidants has been shown to inhibit ER stress, suggesting a reciprocal relationship between ROS and ER stress (109, 110).

Recurrent exposure to oxidative stress may lead a serious damage to the asthmatic airways and the dysregulation of various biological functions (111–118). Heightened oxidative stress could result in mitochondrial dysfunction in asthma (119–121). Notably, ROS and RNS play a major role in airway inflammation and the pathogenesis of asthma (122, 123). Mitochondria closely associate with ER through mitochondrial-associated membranes, which are important for many cellular functions such as the regulation of Ca^2+^ signaling, lipid transport, energy metabolism, and cell survival (124). Activation of different branches of the UPR can interfere with mitochondrial function. For example, ATF4 regulates the ubiquitin ligase Parkin, a crucial regulator of mitochondria function and dynamics (125). ATF6 is also associated with the activation of PCG1α (peroxisome proliferator-activated receptor gamma, coactivator 1 alpha), a master regulator of mitochondrial biogenesis (126). Similarly, enhanced UPR promotes degradation of deSUMOylating enzyme SENP3, leading to enhanced SUMOylated Drp-1 that promotes cytochrome *C* release and caspase-mediated cell death (127). In addition, some of mitochondrial proteins regulate UPR signaling. For instance, mitochondrial fusion protein Mfn2 deficiency leads to enhanced PERK activity and mitochondrial dysfunction. Silencing of PERK results in reduced ROS production, normalized mitochondrial Ca^2+^ and improved mitochondrial morphology, suggesting that PERK is a key regulator of mitochondrial morphology and function (128). Given the close connection between ER and mitochondria, activation of UPR could lead to mitochondrial dysfunction and worsen the disease process in asthma.

## Apoptosis, UPR, and Asthma

Apoptosis is a form of programmed cell death, which cells activate intracellular pathways to terminate themselves in a systematic fashion. Apoptosis occurs as a normal cellular function in response to various stimuli such as virus infection (129). Apoptosis is crucial for development, and for maintaining cellular homeostasis in multicellular organisms. It is considered to be a fundamental pathway of the host innate immune response to virus infections. Failure of a cell to undergo apoptosis, when necessary, leads to several neurological and immunological disorders and infectious diseases (130). Apoptosis is induced by either an extrinsic or intrinsic pathways. The extrinsic pathway is triggered by the binding of death ligands to their receptors such as TNFα and TFNR1, and TNF-related apoptosis-inducing ligand and DR4/5, leading to activation of caspase 9. The intrinsic pathway is initiated by the mitochondria in response to death stimuli generated within the cell, resulting in the release of cytochrome *C* into the cytoplasm (131).

Prolonged activation of ER stress induces apoptosis when adaptive UPR is unable to resolve the ER stress. Chronic non-resolving ER stress has been reported to be a key feature of numerous chronic conditions; such as neurodegenerative disease, diabetes, atherosclerosis, and renal disease (132). In asthma, the non-resolving ER-UPR stress is likely causing chronic activation of IRE1 and CHOP signaling.

Dysregulated apoptosis has been identified as a key driver in asthma pathogenesis. Loss of the normal bronchial pseudostratified epithelium and thickened basement membrane with few basal cells has been reported to be a feature of asthmatic epithelium. Trautmann et al. demonstrated that asthmatic bronchial epithelium is characterized by heightened apoptosis triggered by IFN-γ, and TNF-α secreted by activated eosinophils and T cells. Moreover, TNF receptors, but not Fas, have been shown to play a role in triggering apoptosis in the asthmatic bronchial epithelium (133). Further, this epithelial apoptosis could be associated with a loss of E-cadherin in asthmatic patients (134). Cohen et al. demonstrated increased apoptosis in severe asthmatics but not in mild asthmatics (135). White further suggested that bronchial epithelial cell apoptosis was associated with the chronicity and severity of asthma (136). Therefore, the normal regulation of apoptosis is important for disease control in asthma. However, the ER stress induced apoptosis in asthma is poorly studied. Further studies are required to elucidate the role of ER stress induced apoptosis in different asthma phenotypes.

## Conclusion and Future Directions

**Figure 3:**
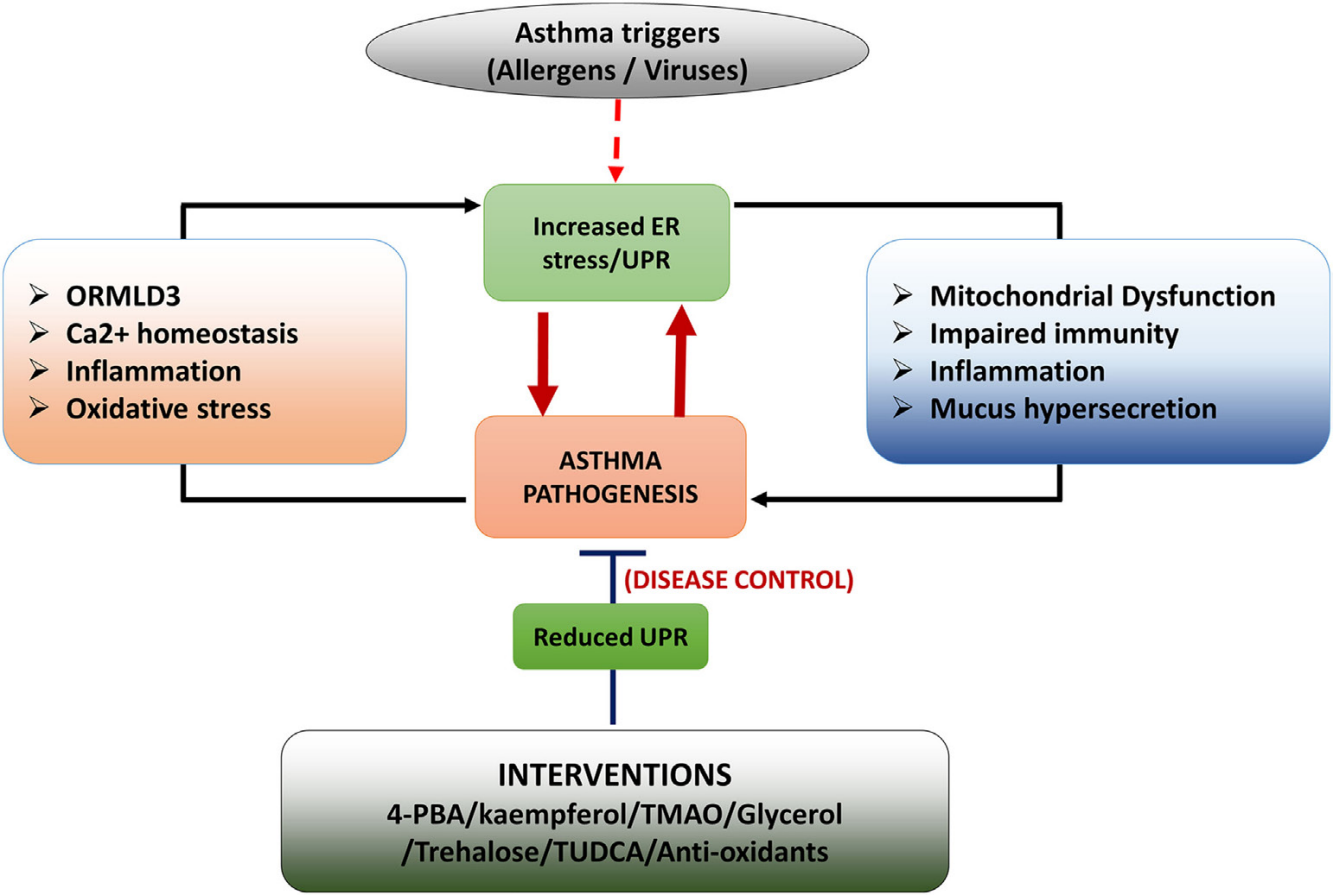
Schematic diagram of role of unfolded protein response (UPR) in asthma development. Asthma triggers such as allergens (HDM) and pathogenic infections (virus infections) induce endoplasmic reticulum (ER) stress/UPR. Activation of UPR signaling triggers asthmatic features in the airways by inducing inflammation, mucus hypersecretion, mitochondrial dysfunction, and impaired innate immunity. Some features of asthmatic airways such as heightened inflammation, oxidative stress, impaired Ca^2+^ signaling and upregulated ORMDL3 expression induce UPR resulting in a positive feedback loop. Interventions such as chemical chaperons and antioxidants increase protein folding and thereby reduce asthmatic features in the airways.

The ER is a dynamic organelle that ensures cellular homeostasis is established in protein translation/folding, inflammation, and apoptosis, particularly in response to allergen and pathogens. The signaling arms of the ER and their sophisticated functions position ER as an important stress sensor and reactor. Abnormal ER function and UPR may well contribute to the disease process in asthma. Asthma is a chronic airways disease with features including heightened inflammation, increased cellular oxidative stress, mucus hypersecretion, and impaired antiviral response. All of these responses may be adversely influenced by ER–UPR in asthma (Figure 3).

It is unclear if UPR signaling is abnormally regulated in asthma, although ORMDL3 has been shown to be increased in asthmatic bronchial epithelial cells, which leads to increased ATF6-mediated ER stress and UPR. Chronic ER stress/UPR could prime the airway cells to be more responsive to allergens or pathogens, leading to exaggerated inflammation, heightened oxidative stress, and reduced antiviral responses. Upon exposure to these stimuli, the activation of the UPR signaling arms may then cause a vicious cycle of continuous UPR–stress–inflammation–mucus production (Figure 3). Impaired antiviral responses would also prime the epithelial environment to be more beneficial for viral replication, which would further increase ER load. When ER fails to regain homeostasis, UPR–apoptosis is then triggered.

While the ER and UPR have been taken the attention of investigators in the areas of diabetes and cancer, the roles of many of these signaling activities in asthma have received less attention. In, particularly how the ER may prime the cellular environment to be hyperresponsive to external stimuli and cause acute exacerbations. Chemical chaperones such as TUDCA and 4-PBA have been shown to have promising effects in reducing ER–UPR and airway inflammation, mucus metaplasia in asthma models. The Food and Drug Administration has approved TUDCA and 4-PBA for the treatment of primary biliary cirrhosis and urea cycle disorders, respectively (137). Small scale clinical trials demonstrate that treatment of TUDCA might have an effect on improving the liver and muscle insulin sensitivity by modulating ER stress (138) and oral 4-PBA treatment provides benefits by alleviating the lipid-induced insulin resistance and β-cell dysfunction caused by ER stress in humans (139). Thus, pharmacological inhibitors based on TUDCA and 4-PBA could be safe potential therapeutic agents for small scale clinical trials for asthma in near future.

In summary, ER stress may play a central role in regulation of pathogenesis of asthma; further investigations of role of ER stress/UPR in asthma would be inevitably helpful to identify potential therapeutic strategies in the asthma development or treatment.

## Author Contributions

PP, AH, and DW designed and wrote the manuscript. PH and LW contributed to revise the manuscript. PW and AH contributed for overall editing, revision, and supervision.

## Conflict of Interest Statement

The authors declare that the research was conducted in the absence of any commercial or financial relationships that could be construed as a potential conflict of interest.
